# Integrated analysis of long non-coding RNAs in human gastric cancer: An in silico study

**DOI:** 10.1371/journal.pone.0183517

**Published:** 2017-08-25

**Authors:** Weiwei Han, Zhenyu Zhang, Bangshun He, Yijun Xu, Jun Zhang, Weijun Cao

**Affiliations:** 1 Department of Gastroenterology, Nanjing First Hospital, Nanjing Medical University, Nanjing, China; 2 General Clinical Research Center, Nanjing First Hospital, Nanjing Medical University, Nanjing, China; University of South Alabama Mitchell Cancer Institute, UNITED STATES

## Abstract

Accumulating evidence highlights the important role of long non-coding RNAs (lncRNAs) in a large number of biological processes. However, the knowledge of genome scale expression of lncRNAs and their potential biological function in gastric cancer is still lacking. Using RNA-seq data from 420 gastric cancer patients in The Cancer Genome Atlas (TCGA), we identified 1,294 lncRNAs differentially expressed in gastric cancer compared with adjacent normal tissues. We also found 247 lncRNAs differentially expressed between intestinal subtype and diffuse subtype. Survival analysis revealed 33 lncRNAs independently associated with patient overall survival, of which 6 lncRNAs were validated in the internal validation set. There were 181 differentially expressed lncRNAs located in the recurrent somatic copy number alterations (SCNAs) regions and their correlations between copy number and RNA expression level were also analyzed. In addition, we inferred the function of lncRNAs by construction of a co-expression network for mRNAs and lncRNAs. Together, this study presented an integrative analysis of lncRNAs in gastric cancer and provided a valuable resource for further functional research of lncRNAs in gastric cancer.

## Introduction

Gastric cancer is the fourth most common cancer and the second leading cause of cancer-related death worldwide [1]. Despite advances in treatment and understanding of the molecular basis of the disease, treatment outcome for gastric cancer still remains undesirable [2]. Long non-coding RNAs (lncRNAs) are a large class of non-protein-coding transcripts that are with more than 200 nucleotide in length. Although without apparent protein coding potential, lncRNAs play critical regulatory roles in a large number of biological processes such as chromatin remodeling, transcription, post-transcriptional processing and intracellular trafficking [3, 4]. It was also reported that lncRNAs played a role in carcinogenesis and could be diagnostic or prognostic biomarkers for gastric cancer. For example, GAPLINC was firstly identified in gastric cancer and its upregulation was associated with shorter survival of gastric cancer patients [5]. Another lncRNA, GClnc1, was up-regulated and associated with tumorigenesis, tumor size, metastasis, and poor prognosis in gastric cancer. Mechanistically, GClnc1 acted as a modular scaffold of WDR5 and KAT2A complexes and specified the histone modification pattern on the target genes, including SOD2 [6]. However, the knowledge of genome scale expression of lncRNAs and their potential biological function in gastric cancer is still lacking.

In the present study, we profiled the global expression patterns and dysregulation of lncRNAs in gastric cancer utilizing RNA-seq and clinical data from 420 gastric cancer patients in The Cancer Genome Atlas (TCGA). We then identified the lncRNAs associated with subtype and prognosis. We also investigate the global relationship between copy number and lncRNA expression. Finally, we inferred the function of lncRNAs with co-expression network analysis.

## Methods

### The Cancer Genome Atlas (TCGA) data

We downloaded RNA-seq data (RNA-seq V2, fastq files) for 420 gastric cancers and 36 adjacent normal tissues from TCGA Data Portal (https://tcga-data.nci.nih.gov/tcga/) and the Cancer Genomics Hub (CGHub, https://cghub.ucsc.edu/). Their related clinical data were obtained on 8 March 2016.

### RNA-seq data processing

LncRNA catalogue was retrieved from GENCODE v19 [7]. The fastq files were aligned to the human reference genome (Ensembl Homo sapiens GRCh37/hg19) using Subread [8] allowing only unique mapping. Using featureCounts [9], the aligned reads were counted on gene-level based on the gene annotation from Ensembl 75 [10] (GENCODE v19).

### Differential expression analysis

Read count tables were imported into the edgeR package [11] and only genes with read count > 0 in at least 75% of the samples were kept for downstream analysis. Then the data were normalized and log-counts per million (log-cpm) were calculated using the voom function of the limma package [12]. The pipeline of the empirical Bayes model implemented by limma was used to identify the differentially expressed genes between two different groups (gastric cancer vs. adjacent normal tissue, diffuse subtype vs. intestinal subtype). Benjamini and Hochberg method was used for adjusting for multiple testing [13]. Genes with adjusted *p*-values below 0.05 and absolute fold change > 2 were considered differentially expressed.

### Additional independent dataset

Additional independent dataset from Korea [14] was downloaded from NCBI Sequence Read Archive (accession number SRP014574). The dataset contained 32 RNA-seq data of gastric cancer and adjacent normal tissue from 16 patients in Korea. Each sample was paired-end sequenced with the Illumina Genome Analyzer II or with the Illumina HiSeq 2000 using HiSeq Sequencing kits. Detailed information about the patients and samples were referenced from the publication by Yoon [14].

### LncRNAs associated with prognosis

Patients with overall survival information available were randomly divided into a training set (n = 259, 66.8%) and a validation set (n = 129, 33.2%). In the training set, univariate Cox proportional hazards regression analysis was employed to explore the associations of different covariates, including lncRNA expression(continuous data), gender, age (cutoff point: median value), grade (1 + 2 vs. 3), histological type (intestinal subtype vs. diffuse subtype), AJCC stage (stage III + IV vs. stage I + II), with patient overall survival. In the case of no death, the event time was censored at the date of last contact. Furthermore, multivariate Cox proportional hazards regression analysis was performed by combining the potential prognostic factors (with *p* values < 0.01 in the univariate Cox regression analysis). *P* < 0.05 was considered statistically significant in the multivariate Cox regression analysis. Then, lncRNAs associated with overall survival were assessed in the validation set. The survival analysis was conducted using the survival package [15].

### LncRNAs in regions of focal copy-number alteration

Copy number segmentation data (level 3) of the Affymetrix SNP 6.0 platform were downloaded from TCGA. GISTIC2.0 [16] was used to identify genomic regions that were focally amplified or deleted. Aberrant regions were considered significant if the assigned FDR *q*-value was less than 0.25. The lncRNAs within these regions were identified using BEDTools [17]. The CNTools package [18] was used to process segmentation data and format the data into a gene-level matrix based on corresponding genomic location of lncRNAs. The correlation between copy number values and the corresponding gene expression levels was estimated using R (Pearson correlation).

### Co-expression network analysis

Voom-transformed read counts of differentially expressed mRNAs and lncRNAs in gastric cancer were used as input to construct signed co-expression networks using the WGCNA package [19] in R. The adjacency matrix was calculated based on pair-wise Pearson correlation coefficients for a signed network. A value of *β* = 7 was chosen as soft-threshold power on the criterion of scale-free topology. Average linkage hierarchical clustering was performed to group genes based on Topological Overlap-based dissimilarity matrix. Modules were identified by using a dynamic tree-cutting algorithm with a minimum module size of 30 genes.

### Gene ontology and pathway enrichment analysis

For the protein-coding genes in each modules, the DAVID bioinformatics database [20] was used to look for enrichment of genes associated with GO biological process terms and KEGG pathways.

## Results

### Differentially expressed lncRNAs in gastric cancer

**Fig 1:**
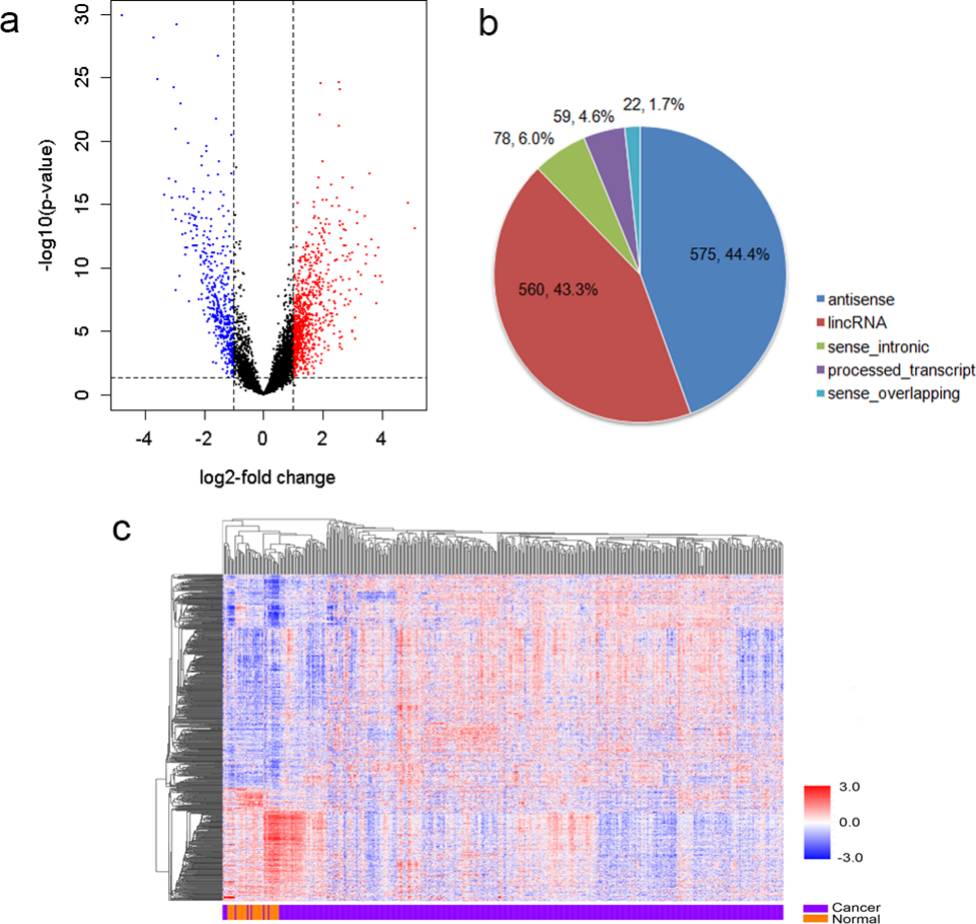
Differentially expressed lncRNAs in gastric cancer. (a) Volcano plot of the differential expression analysis of lncRNAs. (b) Pie charts showing the number of differentially expressed lncRNAs in each category. (c) Heatmap of unsupervised hierarchical clustering of differentially expressed lncRNAs in all samples.

To systematically identify lncRNAs related to gastric cancer, RNA-seq data of 420 gastric cancers and 36 adjacent normal tissues were retrieved from TCGA. After filtering of lowly expressed transcripts, 6,488 lncRNAs were kept for downstream analysis. By the criteria of adjusted *p*-value < 0.05 and absolute fold change > 2, we identified 1,294 lncRNAs differentially expressed in gastric cancer compared with adjacent normal tissues, among which 846 were up-regulated and 448 were down-regulated (Fig 1A and S1 Table). Unsupervised hierarchical cluster analysis revealed two separate clusters between gastric cancer and normal tissues (Fig 1C). We then analyzed the differentially expressed lncRNAs based on their categorizations. The results were indicated in Fig 1B. Antisense transcripts accounted for 44.4%, followed by lincRNAs (43.3%). The remaining non-coding transcript types were sense_intronic transcripts (6.0%), processed_transcripts (4.6%) and sense_overlapping transcripts (1.7%).

### Validation of differentially expressed lncRNAs in additional independent dataset

**Fig 2:**
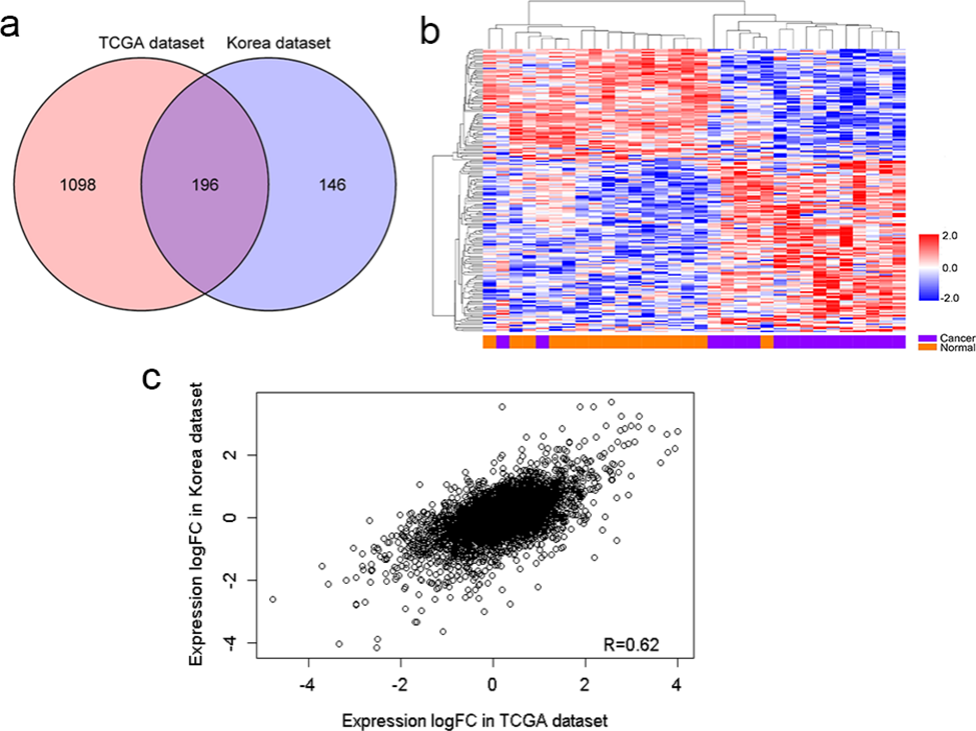
Validation of differentially expressed lncRNAs in additional independent dataset. (a) Venn diagram showing the overlap of differentially expressed lncRNAs in the TCGA dataset versus the Korea dataset. (b) Heatmap of unsupervised hierarchical clustering of differentially expressed lncRNAs in the Korea dataset. (c) Distribution of expression differentials between the TCGA dataset and the Korea dataset.

To provide additional validation of differentially expressed lncRNAs in gastric cancer, we analyzed an independent dataset from Korea [14], which contained RNA-seq data of 16 paired gastric cancers and adjacent normal tissues. The data were processed using the same method as the TCGA dataset. We identified 342 differentially expressed lncRNAs, among which 171 were up-regulated and 171 were down-regulated (S2 Table). The overlap analysis of differentially expressed lncRNAs showed that 196 were overlapped between the Korea dataset and the TCGA dataset and only one gene was in different direction (Fig 2A). Unsupervised hierarchical cluster analysis with overlapped lncRNAs was showed as Fig 2B. Additionally, the distribution of expression differentials between two datasets was significantly concordant (Fig 2C).

### LncRNAs associated with subtype

**Fig 3:**
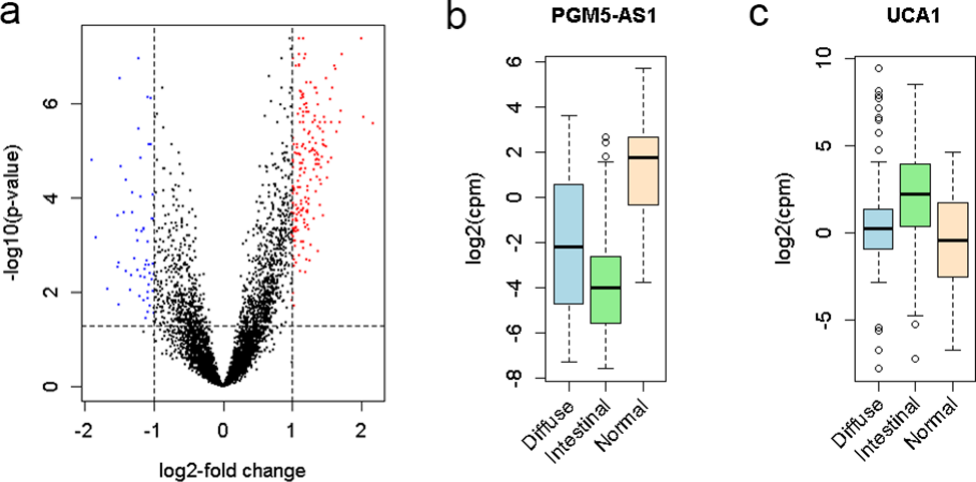
LncRNAs associated with subtype. (a) Volcano plot of the differential expression analysis of lncRNA between the two subtypes. (b) The box plot showing differential expression of PGM5-AS1 among diffuse subtype, intestinal subtype and normal tissue. (c) The box plot showing differential expression of UCA1 among diffuse subtype, intestinal subtype and normal tissue.

Gastric cancer is a heterogeneous disease comprised of two major histological subtypes, intestinal subtype and diffuse subtype [21]. Therefore, we compared lncRNA expression between these two subtypes. We found 192 lncRNAs were up-regulated and 55 were down-regulated in diffuse subtypes compared with intestinal subtypes (Fig 3A and S3 Table). Of the 247 lncRNAs that differentially expressed between two subtypes, 154 lncRNAs were differentially expressed between gastric cancer and adjacent normal tissue. Fig 3B and 3C showed the expression levels of two examples, PGM5-AS1 and UCA1.

### LncRNAs associated with prognosis

**Fig 4:**
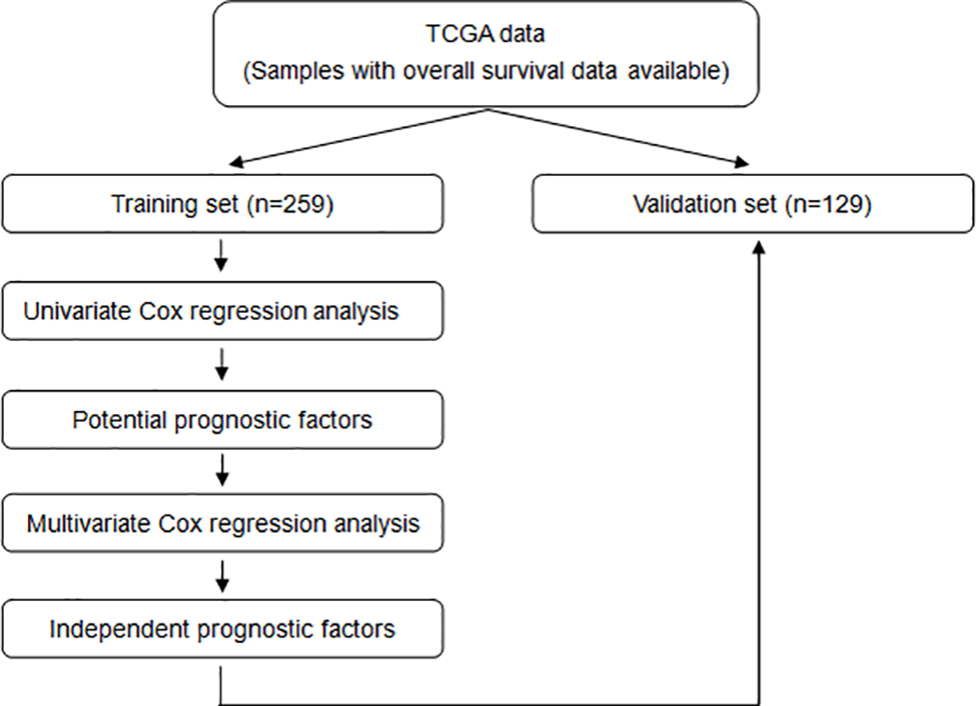
Flow chart of identification of lncRNAs associated with prognosis.

To identify lncRNAs associated with clinical outcome in gastric cancer, patients were divided into a training set and a validation set and survival analyses were performed (Fig 4).In the training set, the univariate Cox regression analysis indicated that AJCC stage (*p* = 0.0027) and 102 lncRNAs were significantly associated with the overall survival of gastric cancer patients. The multivariate Cox regression analysis demonstrated that AJCC stage (*p* = 0.0016) and 33 lncRNAs were independent prognostic factors (Table 1). We then tested whether these 33 lncRNAs were associated with overall survival in the validation set.6 lncRNAs (shown in bold type in Table 1) were validated to be associated with overall survival in the validation set in univariate Cox regression analysis with *p* value < 0.05.

**Table 1:** The list of lncRNAs independently associated with overall survival of gastric cancer.

GeneID	GeneName	GeneBiotype	HR	P.Value	Associated Malignancies
ENSG00000223477	LINC00842	lincRNA	3.0811	4.60E-04	
ENSG00000248664	CTC-498J12.3	antisense	3.8	6.20E-04	
ENSG00000257877	RP3-462E2.3	lincRNA	0.272	1.00E-03	lung adenocarcinoma [22]
ENSG00000228623	ZNF883	lincRNA	2.2771	1.41E-03	
ENSG00000259005	RP3-449M8.6	lincRNA	4.5295	1.97E-03	papillary thyroid cancer [23]
ENSG00000205562	RP11-497E19.1	lincRNA	2.6712	2.01E-03	
ENSG00000249650	RP11-310P5.1	antisense	2.4037	2.10E-03	
ENSG00000265148	BZRAP1-AS1	antisense	0.1808	2.99E-03	nasopharyngeal carcinoma [24]
ENSG00000254985	RSF1-IT2	sense_intronic	0.3269	4.47E-03	
**ENSG00000229431**	RP1-92O14.6	antisense	0.1069	6.25E-03	
ENSG00000232593	LINC01155	lincRNA	0.069	6.33E-03	
**ENSG00000256268**	RP11-221N13.3	lincRNA	0.4815	8.14E-03	oral cancer [25]
ENSG00000227857	RP4-533D7.5	antisense	0.4507	8.32E-03	
ENSG00000251161	RP11-540O11.1	lincRNA	0.3641	1.17E-02	
ENSG00000267583	RP11-322E11.5	lincRNA	0.4109	1.18E-02	
ENSG00000235298	RP11-575L7.8	antisense	3.4252	1.19E-02	
**ENSG00000230107**	CTA-126B4.7	lincRNA	0.4419	1.29E-02	
ENSG00000203441	LINC00449	antisense	0.2775	1.74E-02	
ENSG00000272109	CTD-2260A17.3	antisense	2.4311	1.77E-02	
ENSG00000223396	RPS10P7	lincRNA	4.4906	1.88E-02	
ENSG00000235052	RP1-150O5.3	lincRNA	1.8873	1.98E-02	malignant mesothelioma [26]
ENSG00000242147	RP13-463N16.6	lincRNA	1.7058	2.04E-02	
ENSG00000272707	RP11-534C12.1	lincRNA	0.3848	2.16E-02	
ENSG00000157152	SYN2	processed_transcript	1.811	2.33E-02	
ENSG00000267493	CIRBP-AS1	antisense	5.2374	2.48E-02	lung cancer [27]
ENSG00000227914	RP11-130C19.3	antisense	0.5584	3.53E-02	
**ENSG00000270605**	RP5-1092A3.4	antisense	2.8997	3.65E-02	
ENSG00000232710	RP4-669P10.16	sense_intronic	2.3053	3.87E-02	
ENSG00000267649	CTD-2587H24.10	lincRNA	0.4847	4.49E-02	
ENSG00000229656	RP11-462L8.1	lincRNA	2.1174	4.70E-02	
**ENSG00000271816**	RP11-574K11.28	processed_transcript	0.3661	4.74E-02	
**ENSG00000228214**	LINC00693	antisense	0.5436	4.92E-02	pancreatic ductal adenocarcinoma [28]
ENSG00000224066	RP4-622L5.7	antisense	0.3485	4.99E-02	

HR: hazard ratio.

Bold type indicates lncRNAs validated to be associated with overall survival in the validation set.

### LncRNAs in regions of somatic copy number alterations

**Fig 5:**
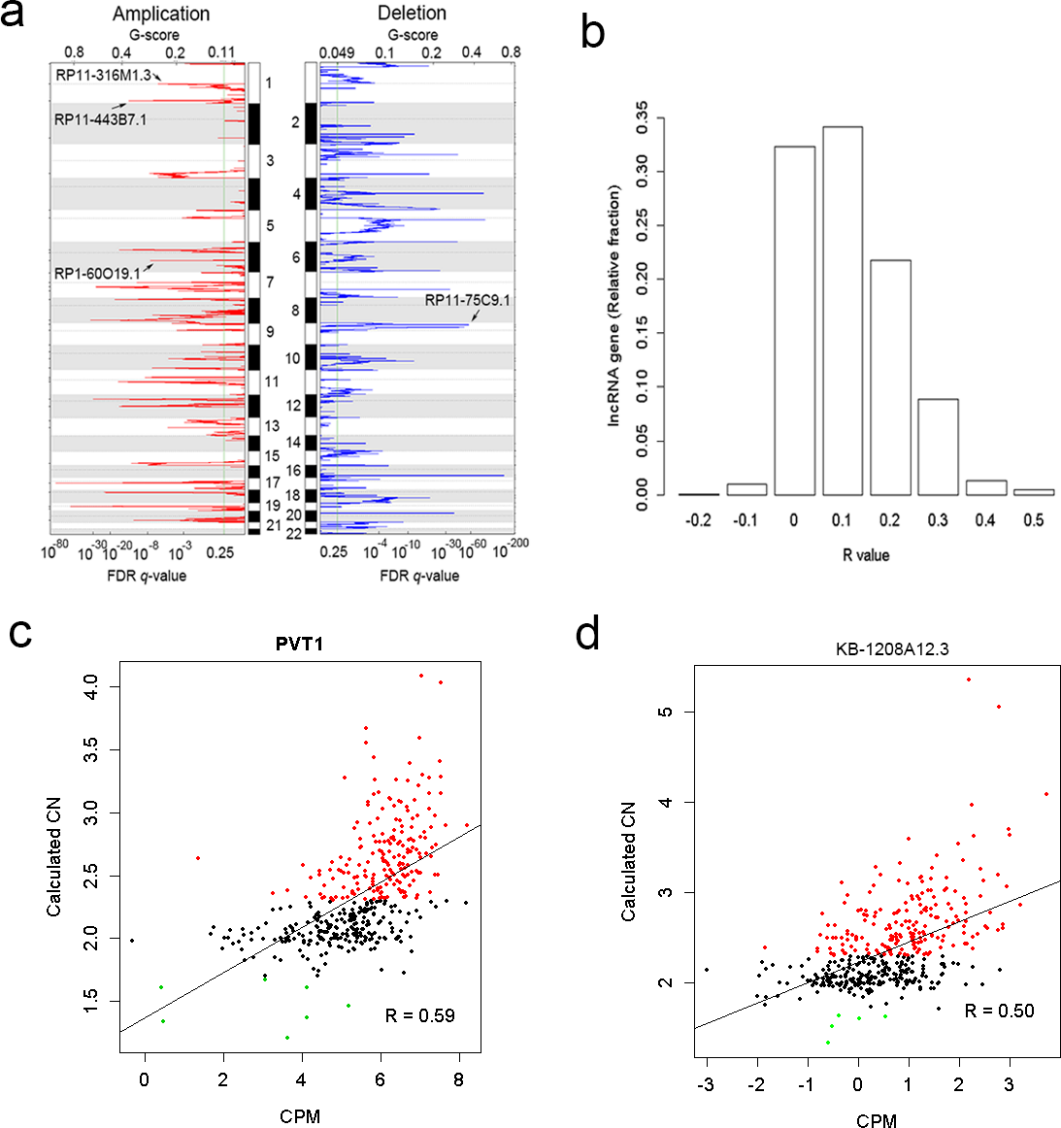
LncRNAs in regions of somatic copy number alterations. (a) Chromosomal view of amplification and deletion peaks of gastric cancer. (b) Histogram of correlations between copy number and RNA expression level. (c) The correlation between copy number and expression level of PVT1. (d) The correlation between copy number and expression level of KB-1208A12.3.

To characterize the focal somatic copy number alterations (SCNAs) that harbor differentially expressed lncRNAs in gastric cancer, GISTIC2.0 [16] was used to identify genomic regions that were focally amplified or deleted. Then the lncRNA-containing loci were mapped to these focal alteration regions. We found 181 differentially expressed lncRNAs located in the recurrent SCNA regions (Fig 5A and S4 Table).

To estimate the contribution of SCNAs to lncRNA dysregulation in gastric cancer, we analyzed the correlation between copy number and RNA expression level for differentially expressed lncRNAs (Fig 5B). 32.42% of the lncRNAs were shown positive correlations (R > 0.2) between their copy numbers and RNA expression levels. For example, PVT1 and KB-1208A12.3 had a pattern of increased expression driven by copy number amplication (Fig 5C and 5D).

### Co-expression network analysis

**Fig 6:**
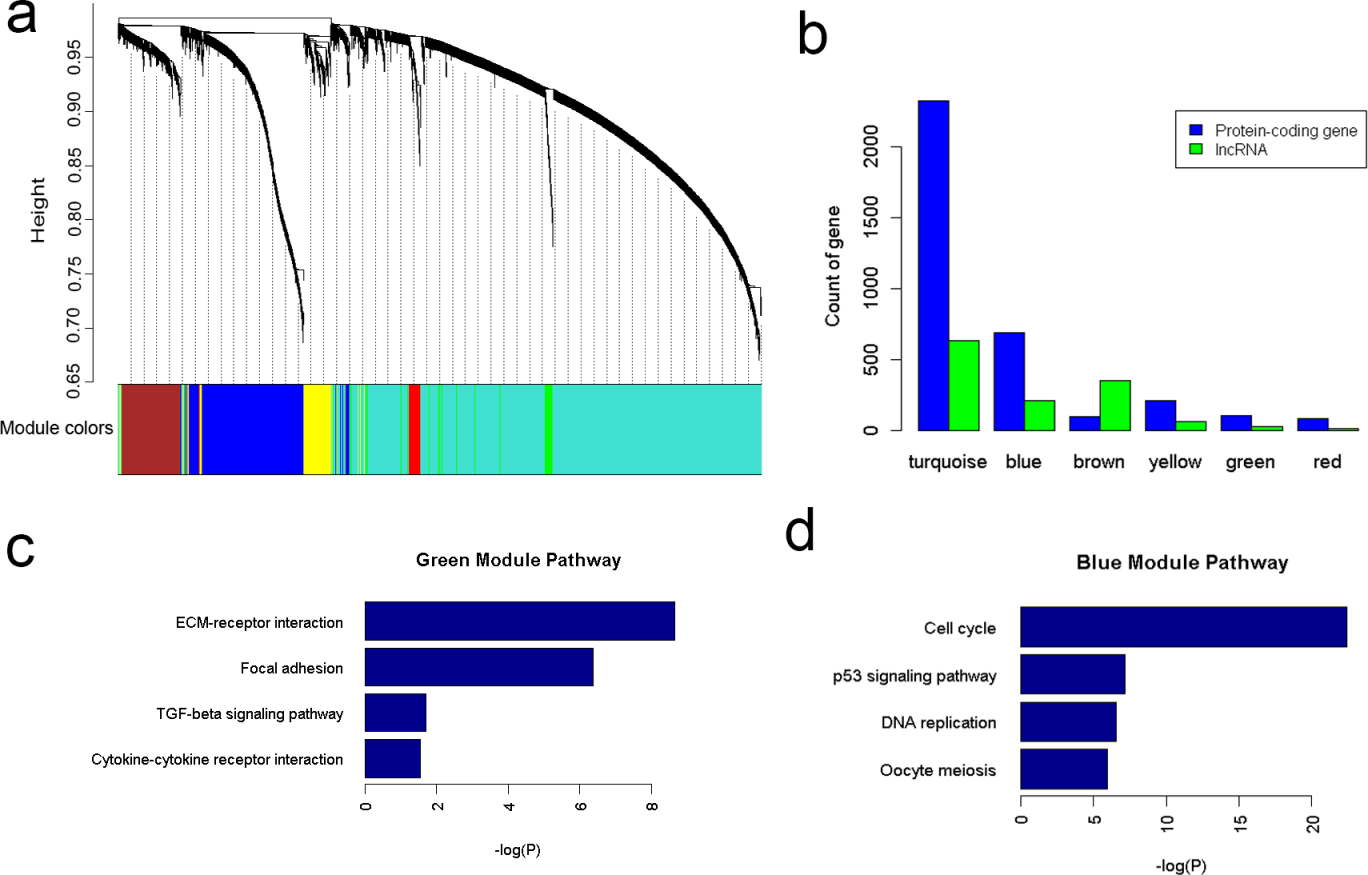
Co-expression network analysis. (a) Gene clustering and module identification. (b) LncRNAs distribution in the different modules. (c) Pathway analysis of genes in green module. (d) Pathway analysis of genes in blue module.

To illustrate the function of lncRNAs, we constructed a co-expression network for both mRNAs and lncRNAs that were identified as differentially expressed in gastric cancer. Using WGCNA [19], we clustered highly co-expressed genes into 6 co-expression modules (Fig 6A and S5 Table). The module size ranged from 96 to 2,951 genes. The percentage of lncRNAs in each module ranged from 13.54% (red module) to 78.92% (brown module) (Fig 6B). The turquoise module had both the highest number of genes and of lncRNAs (2,951 genes of which 629 lncRNAs).

KEGG pathway analyses and Gene ontology (GO) enrichment analyses were performed on each single module (S6 Table). Notably, pathway analysis showed that genes in green module were highly enriched in the activated pathways such as ECM-receptor interaction, focal adhesion and TGF-β signaling pathway (Fig 6C). Genes in the blue module were significantly enriched in cell cycles, p53 signaling pathway and DNA replication (Fig 6D).

## Discussion

Here, we comprehensively analyzed the expression patterns of lncRNAs in gastric cancer using RNA-seq data from TCGA. We identified more than 1,000 lncRNAs differentially expressed in gastric cancer. As expected, we validated some known gastric cancer related lncRNAs, such as HOTAIR [29], PVT1 [30], GAPLNC [5], et al, which giving us confidence in our methodology. Also, we identified a large number of lncRNAs that had not been previously reported in gastric cancer. To functionally characterize these lncRNAs may substantially expand our understanding of the molecular mechanisms involved in gastric cancer pathogenesis.

We compared lncRNA expression between two major subtypes of gastric cancer, intestinal subtype and diffuse subtype. It was reported that there are underlying biologic and genomic distinctions between these two subtypes [31, 32]. Our study identified hundreds of lncRNAs showing significant subtype-specific expression patterns, which may have an important function in individual subtypes.

To identify lncRNAs with potential biological function, we analyzed the correlation between the expression levels of lncRNAs and patient overall survival using univariate and multivariate Cox regression analyses. In the training set, we identified 33 prognosis-associated lncRNAs in gastric cancer. Some of them were reported to be associated with malignancies. 6 lncRNAs were validated to be associated with overall survival in the internal validation set. However, the prognostic significance of these lncRNAs needs further investigation in an independent patient cohort. Our analysis missed some lncRNAs that have reported to be associated with overall survival of gastric cancer patients, such as GAS5 [33] and HOTAIR [34]. We think it was due to the different distributions of the patient populations in terms of age, gender, histological subtype, stage, etc.

It has been suggested that cancer driver genes are often located in the SCNAs [35]. Previous studies have identified lncRNAs as drivers such as GAPLINC [5] and FAL1 [36]. Here, we identified 181 differentially expressed lncRNAs located in the recurrent SCNA regions. Furthermore, we identify lncRNAs which were shown positive correlations between their RNA expression levels and their gene copy numbers. For example, we found PVT1 had a pattern of increased expression driven by copy number amplication, which has been validated in a previous study [37]. We anticipated further function analysis of the lncRNAs associated with SCNAs will help to find driver lncRNAs in gastric cancer.

To illustrate the function of lncRNAs, we constructed a co-expression network for mRNAs and lncRNAs. Pathway analysis revealed genes in green module were enriched in ECM-receptor interaction, focal adhesion and TGF-β signaling pathway. It is reported these pathways played critical roles in cancer invasion and metastasis [38, 39]. So lncRNAs in this module may be involved in invasion and metastasis of gastric cancer. Interestingly, RP11-838N2.4 (also known as GAPLINC [5]) and LINC00152 [40], two lncRNAs in green module, were both reported to be associated with invasion of gastric cancer. Genes in the blue module were significantly enriched in cell cycles, p53 signaling pathway and DNA replication. It is well known that p53 signaling pathway plays the central role in maintenance of genomic stability by acting through cell-cycle arrest, senescence, and apoptosis [39, 41]. Of note, two of most well-known oncogenic lncRNAs HOTAIR [29] and PVT1 [30] were in this module. So we inferred that lncRNAs in the blue module may play important roles in gastric cancer.

Taken together, we presented an integrative analysis of lncRNAs in gastric cancer. We identified a panel of dysregulated lncRNAs that may be potential drivers and diagnostic, therapeutic biomarkers of gastric cancer, although targeted validation of these lncRNAs is still needed. This study provided a valuable resource for further functional research of lncRNAs in gastric cancer.

## Supporting information

S1 Table Differentially expressed lncRNAs in gastric cancer. (XLSX)

S2 Table Differentially expressed lncRNAs of Korea dataset. (XLSX)

S3 Table Differentially expressed lncRNAs between intestinal subtype and diffuse subtype. (XLSX)

S4 Table Differentially expressed lncRNAs located in the recurrent SCNA regions. (XLSX)

S5 Table Co-expression network analysis using WGCNA. (XLSX)

S6 Table KEGG pathway analyses and GO enrichment analyses of each single module. (XLSX)
